# Potent Inhibition of Late Stages of Hepadnavirus Replication by a Modified Cell Penetrating Peptide

**DOI:** 10.1371/journal.pone.0048721

**Published:** 2012-11-16

**Authors:** Fabien Abdul, Bénédicte Ndeboko, Thierry Buronfosse, Fabien Zoulim, Michael Kann, Peter E. Nielsen, Lucyna Cova

**Affiliations:** 1 Université de Lyon 1, Lyon, France; 2 Institut National de la Santé et de la Recherche Medicale (INSERM) U1052, Centre de Recherche en Cancérologie de Lyon (CRCL), Lyon, France; 3 CNRS UMR 5286, Centre de Recherche en Cancérologie de Lyon, Lyon, France; 4 VetAgro-Sup, Marcy l'Etoile, France; 5 Université de Bordeaux, Microbiologie Fondamentale et Pathogénicité, UMR 5234, Bordeaux, France; 6 CNRS, Microbiologie fondamentale et Pathogénicité, UMR 5234, Bordeaux, France; 7 CHU de Bordeaux, Bordeaux, France; 8 Department of Cellular and Molecular Medicine and Department of Drug Design and Pharmacology, Faculty of Health and Medical Sciences, The Panum Institute, University of Copenhagen, Copenhagen N, Denmark; Yonsei University, Republic of Korea

## Abstract

Cationic cell-penetrating peptides (CPPs) and their lipid domain-conjugates (CatLip) are agents for the delivery of (uncharged) biologically active molecules into the cell. Using infection and transfection assays we surprisingly discovered that CatLip peptides were able to inhibit replication of Duck Hepatitis B Virus (DHBV), a reference model for human HBV. Amongst twelve CatLip peptides we identified Deca-(Arg)_8_ having a particularly potent antiviral activity, leading to a drastic inhibition of viral particle secretion without detectable toxicity. Inhibition of virion secretion was correlated with a dose-dependent increase in intracellular viral DNA. Deca-(Arg)_8_ peptide did neither interfere with DHBV entry, nor with formation of mature nucleocapsids nor with their travelling to the nucleus. Instead, Deca-(Arg)_8_ caused envelope protein accumulation in large clusters as revealed by confocal laser scanning microscopy indicating severe structural changes of preS/S. Sucrose gradient analysis of supernatants from Deca-(Arg)_8_-treated cells showed unaffected naked viral nucleocapsids release, which was concomitant with a complete arrest of virion and surface protein-containing subviral particle secretion. This is the first report showing that a CPP is able to drastically block hepadnaviral release from infected cells by altering late stages of viral morphogenesis *via* interference with enveloped particle formation, without affecting naked nucleocapsid egress, thus giving a view inside the mode of inhibition. Deca-(Arg)_8_ may be a useful tool for elucidating the hepadnaviral secretory pathway, which is not yet fully understood. Moreover we provide the first evidence that a modified CPP displays a novel antiviral mechanism targeting another step of viral life cycle compared to what has been so far described for other enveloped viruses.

## Introduction

Throughout the last decade cationic cell-penetrating peptides (CPPs) such as Tat, Penetratin, or oligoarginines, have been identified and characterized by their ability to be internalized by mammalian cells [1], [2], [3], [4]. CPPs are short cationic peptides of 5–40 amino acids, with the ability to pass the lipophilic barrier of the cellular membranes by yet not fully elucidated receptor-independent mechanisms, although endocytotic pathways seem to play a major role for most of the CPPs. They also have the capacity to promote cellular delivery of covalently or noncovalently conjugated bioactive cargoes such as peptides, peptide nucleic acids (PNA) and proteins into cells. Clinical applications have been suggested for the transfer of antibiotics and other drugs [5], [6], [7]. Moreover, it has been found that CPPs display a broad range of antibacterial, antifungal, antiviral, or even antitumoral activities [8], [9], [10], [11], [12]. Antiviral activities of cationic peptides were shown to be related to the interference with viral adsorption and entry process or are a result of a direct effect on the viral envelope [10]. Such antiviral activities were reported for different enveloped viruses (*e.g*., HIV, HSV and VSV) [10].

Examples of CPPs inhibiting Hepatitis B Virus (HBV) replication have not yet been reported. HBV is a prototype member of the hepadnavirus family, which comprises mammalian and avian hepadnaviruses. All members share a common replication strategy and the Duck Hepatitis B Virus (DHBV), avian hepadnavirus, is frequently used as a model in pharmacological investigations. HBV is a small enveloped virus with a partially double stranded DNA genome. It causes acute and chronic liver infection. Chronic HBV infection represents a major public health problem with 350 million chronically infected individuals [13]. Chronic HBV infection is the major cause of liver cirrhosis and hepatocellular carcinoma in numerous regions of the world [14] leading to 600,000 deaths per year. Present therapeutic agents for HBV address either the host immune system (α-interferon) or inhibit viral reverse transcription (*e.g*. the nucleoside inhibitors Lamivudine (3TC), Adefovir, Entecavir). Both therapies have, however, severe side effects and their efficacy is either partial with less than 30% (interferon) [15] or counteracted by the development of resistances. Moreover the current antiviral treatments do not eliminate the covalently closed circular viral DNA (cccDNA), a viral minichromosome that is responsible for the persistence of infection. Therefore, alternative therapeutic approaches for chronic hepatitis B are highly warranted.

The life cycle of HBV begins with the binding of HBV virions to surface receptors, which are not unequivocally identified yet. Internalization is realized by endocytosis without posttranslational modification of virus interior or acidification [16]. The genome-containing capsid becomes released into the cytoplasm, migrates via microtubules to the nucleus [17] into which the viral DNA becomes released [18]. Subsequently, the genome is repaired by cellular polymerases to a covalently closed circular DNA (cccDNA), which stays episomal [19]. The cccDNA is the template for viral messenger RNA (mRNA) synthesis including the viral pregenomic RNA (pgRNA) [19], [20]. The mRNAs are exported unspliced to the cytoplasm where translation occurs. The pgRNA encodes for the viral polymerase and the core protein. Two hundred forty copies of the core protein assemble to the viral capsid into which the viral polymerase attached to the encapsidation signal ε on the pgRNA become encapsidated. The pgRNA is then reverse transcribed followed by synthesis of an incomplete second strand (RC-DNA) [19], [21]. These mature capsids have two possible fates: early in infection they transport the progeny genome to the nucleus leading to cccDNA amplification [21], [22]. Later in infection they attach to the viral surface proteins leading to secretion of progeny virions. In contrast to HIV, JV and HSV where interaction with the surface proteins takes place at the plasma membrane, HBV capsid – surface protein interaction occurs at the preGolgi compartment. Like many other viruses (retroviruses and some RNA viruses) externalization requires multivesicular bodies despite of intracellular interaction with surface proteins [23]. The viral surface proteins are synthesized in excess to the number of molecules needed for virus formation. The super numerous surface proteins form subviral particles, which leave the cell by secretion without the need of multivesicular body formation.

It is well known in cell culture experiments (in particular after transfections) that some conditions also favor the secretion of nude capsids, which are not surrounded by the viral surface proteins [23], [24], [25]. These capsids leave the cells by budding, probably at the plasma membrane involving Alix protein and independently of the ESCRT complex [26].

Since HBV has an extremely narrow host range infecting only humans and chimpanzee, the closely related DHBV represents a reference and a very useful model to analyze gene function, viral replication and to evaluate novel antiviral strategies [27], [28], [29], [30], [31], [32], [33]. In search for new anti-HBV approaches, we have investigated in this model the ability of Peptide Nucleic Acids (PNAs) targeting the HBV signal ε to inhibit viral reverse transcription. PNAs, a third generation antisense agents, are uncharged molecules, and covalent conjugation to CPPs improves their cellular uptake [34]. We have previously reported in the DHBV infection model that conjugation of a PNA targeting virus ε to a CPP efficiently inhibited viral replication in primary duck hepatocyte culture [35]. Recent reports showed that the cellular bioavailability of CPP-PNAs could be considerably further improved by chemical conjugation to a lipid domain such as a fatty acid [36]. Thus, such CatLip peptides are a new family of drug delivery conjugates that can enhance both cellular uptake as well as endosome escape of the PNA conjugate. We were initially interested in testing such PNA-CatLip conjugates for their ability to inhibit hepadnaviral replication using the DHBV model. Surprisingly, in a pilot study using as a negative control a CatLip alone, in the absence of its PNA cargo, we observed inhibition of DHBV replication.

This intriguing observation prompted us to test a series of CatLip peptides for their ability to inhibit hepadnaviral replication *in vitro*, using the DHBV infection model, in virus-infected primary duck hepatocytes (PDH) or in previously described stably DHBV-transfected LMH-D2 cells [37], [38]. Using different fatty acid domains and oligo-arginine lengths we identified Decanoyl-(Arg)_8_ ((Deca-(Arg)_8_) as the most effective CatLip inhibiting DHBV release in virus-infected PDH or LMH-D2 cells. We further identified the secretion step in which Deca-(Arg)_8_ interfered with DHBV replication. We provide here the first evidence that such modified CPP can drastically alter hepadnaviral secretion without interfering with viral adsorption or entry process as demonstrated for other enveloped viruses. Moreover this is the first report demonstrating that a modified CPP can alter viral morphogenesis and block subviral particle and viral particle release, targeting yet another step of viral life cycle compared to what has been described for other viruses.

## Results

### Inhibition of DHBV Secretion by Different CatLip

**Figure 1:**
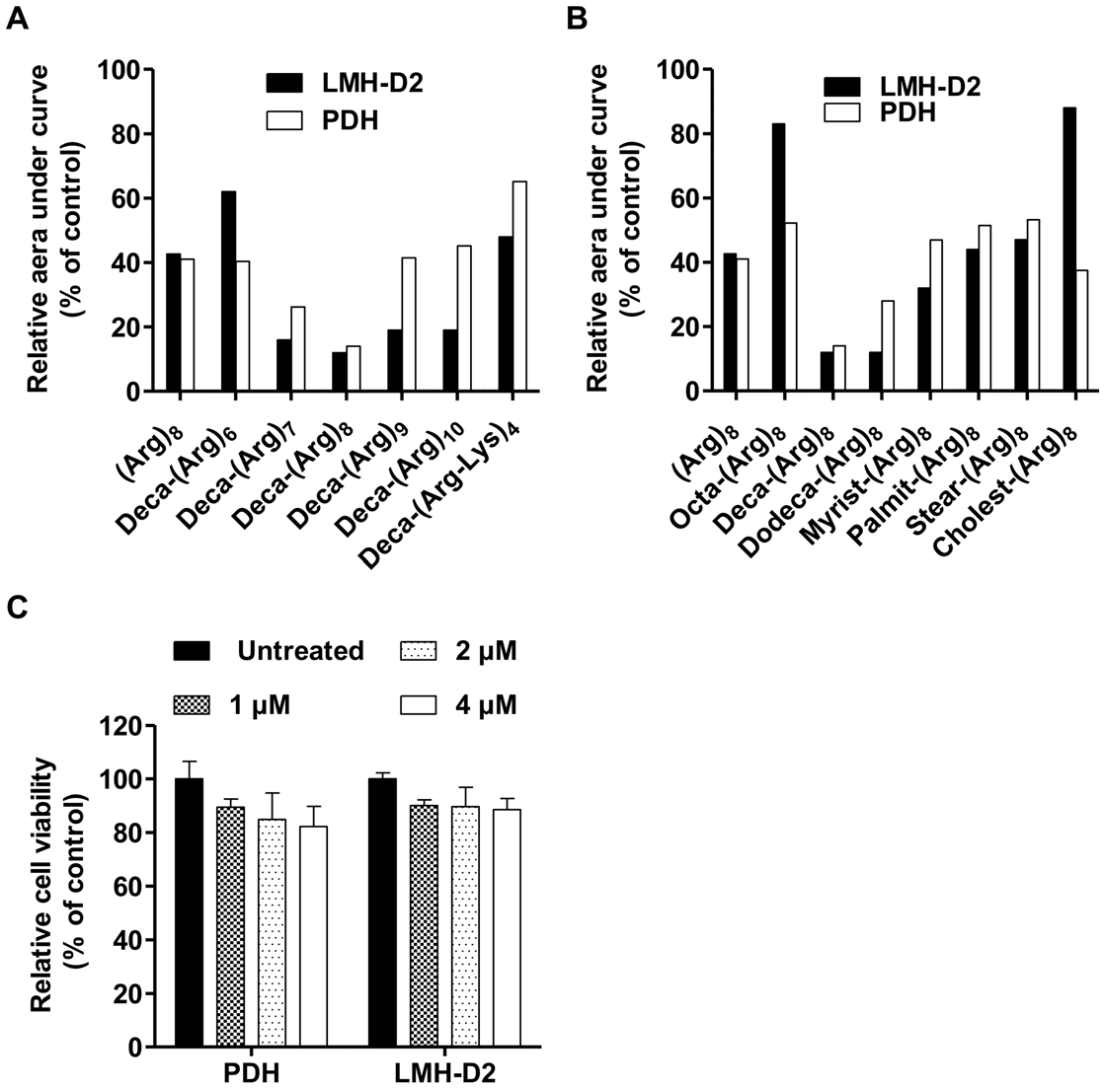
Inhibition of DHBV release in PDHs and LMH-D2 cells by modified cationic peptides and effect of Deca-(Arg)_8_ on cell viability. Infected PDH and LMH-D2 cells were treated with 2 µM of several modified cationic peptides for 5 days. Cells supernatants were collected daily and were spotted onto positively charged nylon membrane and DHBV DNA was detected by hybridization with a DHBV DNA probe labeled with ^32^P, and then was quantified by PhosphorImager scanning using ImageQuant software (Molecular Dynamics) to monitor DHBV release. Relative areas under curves determined by the means of duplicate, compared to untreated cells set at 100% are represented. The two representations show the effect of modified cationic peptides derived from (Arg)_8_ sequence with increasing number of arginine in the peptide (**A**) or with increasing number of carbon in the fatty acid chain (**B**). Dose-dependent effects of Deca-(Arg)_8_ transduction on cell viability (**C**). Cell viability was determined by enzymatic activity MTT assay after daily incubation with different concentrations of Deca-(Arg)_8_ ranking from 1 µM to 4 µM during six days. The error bars display the standard deviation of duplicates in three independent experiments.

To investigate whether different CatLip conjugates could inhibit DHBV replication, a series of oligo-arginines-based CatLip were synthesized and evaluated for their antiviral activity against DHBV (Table 1). All tested CatLip led to a decrease in DHBV secretion in both PDH and LMH-D2 cells with IC_50_ ranking from 0.6 to 2.8 µM (Table 1). By varying the number of arginines in the polycationic domain and the number of carbon atoms in the fatty acid chain, the decanoyl derivatized octa-arginine, Deca-(Arg)_8_, was identified as the most efficient inhibitor of DHBV secretion in both PDH and LMH-D2 cells, leading to a decrease of viral release by 86% and 88%, respectively, as compared to the untreated controls (Table 1, Fig. 1A–B). Based on these results we chose Deca-(Arg)_8_ for further studies of the impact of CatLip on DHBV replication.

**Table 1:** CPP sequences and IC50 of DHBV release.

		IC_50_ (µM)	
CPPs	Sequences	PDH	LMH-D2
(Arg)_8_	H-D(WRRRRRRRRG)-NH_2_	1.8	1.3
Octa-(Arg)_8_	Octanoic acid (C8:0)-D(WRRRRRRRRG)-NH_2_	1.7	2.7
Deca-(Arg)_6_	Decanoic acid (C10:0)-D(WRRRRRRG)-NH_2_	1.4	2.2
Deca-(Arg)_7_	Decanoic acid (C10:0)-D(WRRRRRRRG)-NH_2_	0.9	0.8
Deca-(Arg)_8_	Decanoic acid (C10:0)-D(WRRRRRRRRG)-NH_2_	0.6	0.3
Deca-(Arg)_9_	Decanoic acid (C10:0)-D(WRRRRRRRRG)-NH_2_	1.6	0.9
Deca-(Arg)_10_	Decanoic acid (C10:0)-D(WRRRRRRRRG)-NH_2_	1.4	0.7
Dodeca-(Arg)_8_	Dodecanoic acid (C12:0)-D(WRRRRRRRRG)-NH_2_	0.9	0.7
Myrist-(Arg)_8_	Myristic acid (C14:0)-D(WRRRRRRRRG)-NH_2_	1.2	1.2
Palmit-(Arg)_8_	Palmitic acid (C16:0)-D(WRRRRRRRRG)-NH_2_	1.8	1.7
Stear-(Arg)_8_	Stearic acid (C18:0)-D(WRRRRRRRRG)-NH_2_	1.9	1.8
Cholest-(Arg)_8_	Cholesteryl(C31)-RRRRRRRRG-NH_2_	1.8	2.8
Deca-(Arg-Lys)_4–4_	Decanoic acid (C10:0)-RRRRKKKKG-NH_2_	2.3	1.9
(Arg)_8_-FITC	Fluoresceinyl-lysine-RRRRRRRRG-NH_2_	ND	
Deca-(Arg)_8_-FITC	Decanoic acid (C10:0)-Fluoresceinyl-lysine–RRRRRRRRG-NH_2_	ND	

Sequences are shown in uppercase letters for peptides. The amino acids are **D** amino acids isomers. The carbohydrates chains are shown in lowercase letters. IC_50_ represent the concentration of CPP resulting in 50% inhibition of DHBV release in PDH and LMH-D2 cells.

To examine toxic effects of the Deca-(Arg)_8_ peptide on PDH and LMH-D2 cells, the cells were exposed daily (concentrations ranking from 1 µM to 4 µM) to Deca-(Arg)_8_ for 6 days and cell viability was assayed by MTT test. No statistically significant difference was found between the viability of control (untreated) cells and peptide-exposed cells, indicating that inhibition of DHBV secretion was not associated with Deca-(Arg)_8_ cytotoxicity (Fig. 1C).

**Figure 2:**
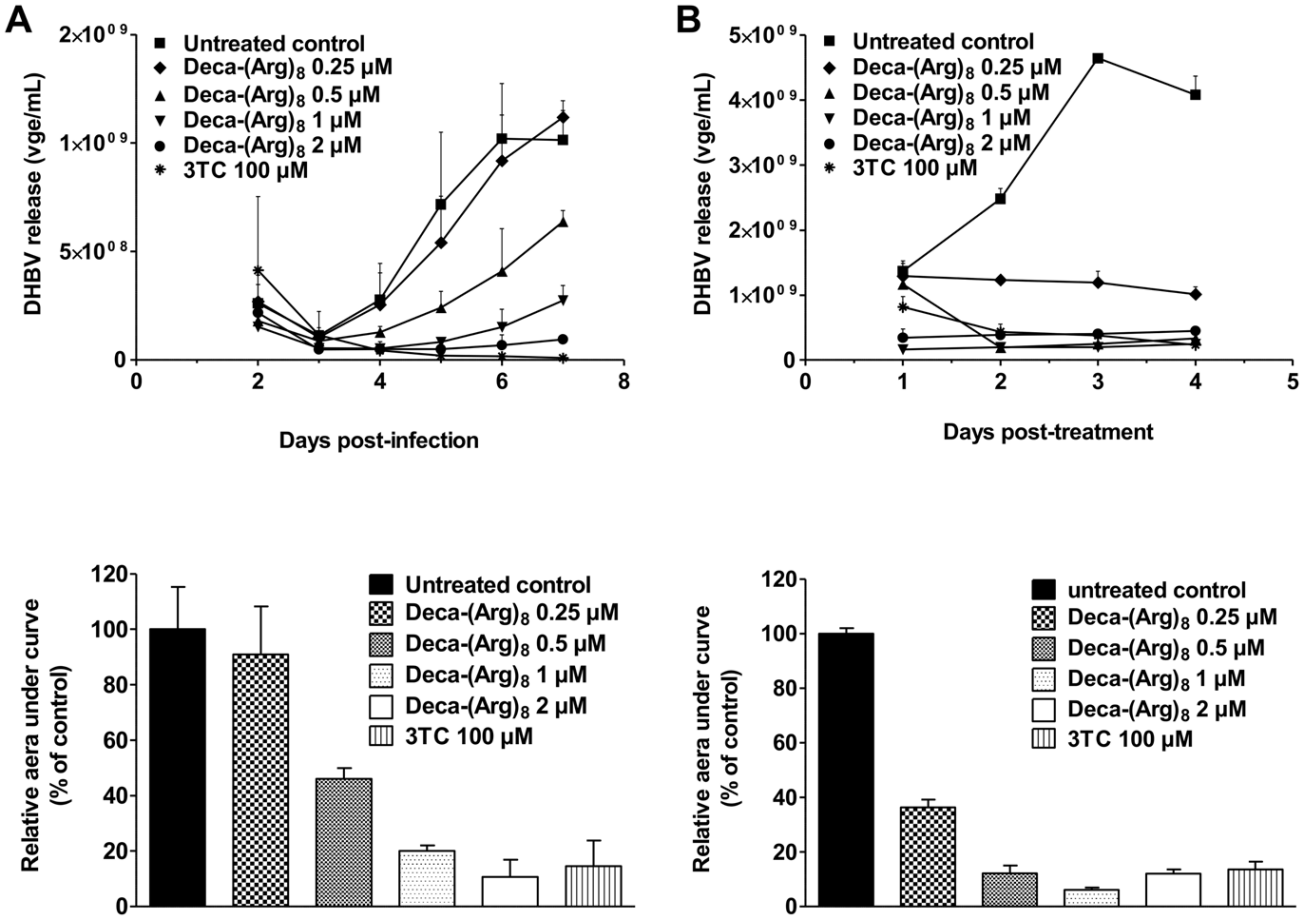
Dose dependent inhibition of hepadnaviral release by Deca-(Arg)_8_ in different cell culture systems. DHBV-infected PDHs (**A**), and stable transfected LMH-D2 cells (**B**) were treated with increasing amounts of Deca-(Arg)_8_ ranking from 0.25 µM to 2 µM in duplicates for 6 and 4 days, respectively. Cell culture supernatants were collected daily during treatment. The kinetics of viral release in cell culture supernatants, monitored by dot-blot hybridization and quantified by PhosphorImager scanning using ImageQuant software (Molecular Dynamics) is represented on the upper panel. Relative areas under curves determined by the means of duplicate, compared to untreated cells, are represented in the lower panel. The curves are representative of at least two independent experiments.

To further explore the antiviral effect of Deca-(Arg)_8_, we investigated the kinetics of DHBV release following administration of different peptide doses. Deca-(Arg)_8_ was administrated daily at 0.25 to 2 µM to LMH-D2 and PDH cells during 4 and 6 days, respectively, and the impact on viral release was monitored in cell culture supernatants. The results shown in Figure 2 confirmed that Deca-(Arg)_8_ treatment induced a strong and dose-dependent decrease in DHBV release in both PDH and LMH-D2 cells. Thus 2 µM dose of Deca-(Arg)_8_ led to a decrease in DHBV secretion reaching 90% at day 7, showing a similar inhibition efficiency as 3-TC at 100 µM (Fig. 2). To test whether the peptide Deca-(Arg)_8_ may also block HBV release, we have evaluated the effect of Deca-(Arg)_8_ peptide on HBV release in stably HBV-transfected cell line HepG2.2.15 (constitutively secreting virus). Deca-(Arg)_8_ was administrated daily at 0. 5 to 4 µM to HepG2.2.15 during 5 days, and the impact on viral release was monitored in cell culture supernatants by dot-blot. Thus, we observed a dose-dependent inhibition of HBV release up to 50% with 4 µM of Deca-(Arg)_8_ in supernatants of HepG2.2.15 (Supplementary data Fig. S1).

To analyze whether antiviral activity is caused solely by the alkyl chain or by the cationic peptide, a 5-days treatment with (Arg)_8_ (0.5 to 2 µM) or decanoic acid (from 0.5 to 10 µM) was performed. While decanoic acid alone was unable to significantly decrease DHBV secretion, (Arg)_8_ treatment by contrast led to a dose-dependent inhibition of DHBV secretion, although at considerably lower inhibition efficacy as compared to Deca-(Arg)_8_ (i.e. 60% versus 90% of inhibition, Supplementary data Fig. S2).

### Internalization of Deca-(Arg)_8_

**Figure 3:**
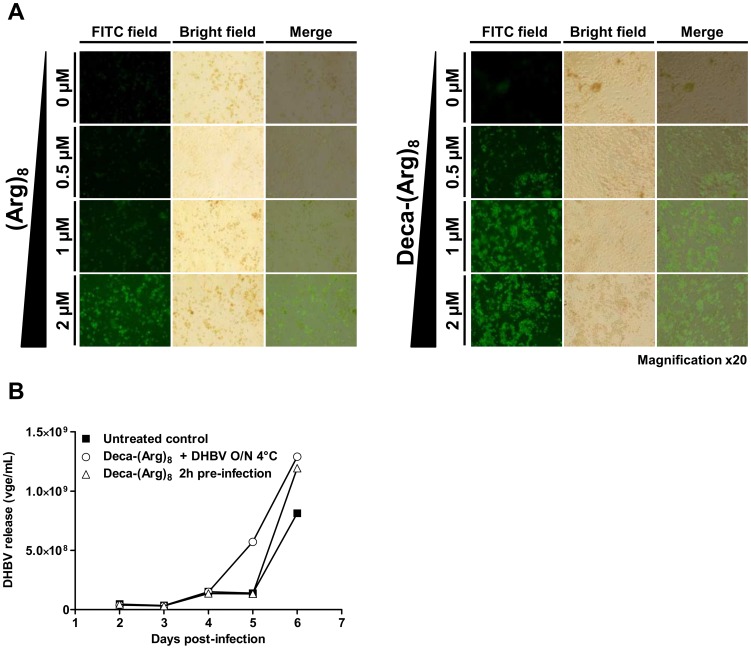
Effect of Deca-(Arg)_8_ on PDHs and DHBV. (**A**); PDHs were plated and treated with (Arg)_8_ (left panel) or Deca-(Arg)_8_ (right panel) coupled to FITC at different concentration ranking from 0.5 µM to 2 µM and cells were incubated at 37°c, 5% of CO_2_ for 24 hours. Cells were observed by fluorescence microscopy (Magnificationx20). (**B**); PDH were plated and infected with DHBV. PDHs were either treated with 2 µM of Deca-(Arg)_8_, washed 2 hours later and then were infected with DHBV or before infection the DHBV inoculum was treated overnight with 2 µM of Deca-(Arg)_8_ and was use for infection of PDHs. The release of viral particles in cell culture supernatants was monitored by dot-blot hybridization and was quantified by PhosphorImager scanning using ImageQuant software (Molecular Dynamics).

Next we analyzed cellular internalization of Deca-(Arg)_8_-FITC and Arg_8_-FITC in PDH cells using fluorescence microscopy. The data presented in Figure 3A shows that both (Arg)_8_-FITC and Deca-(Arg)_8_-FITC were internalized in a dose-dependent manner. From a concentration of 0.5 µM onwards a higher number of FITC-labelled cells were observable using Deca-(Arg)_8_-FITC while Arg_8_-FITC treated cells remained negative, indicating an increased efficiency of internalization. This difference was even more striking at 2 µM, where 70% of FITC-labelled cells were (Arg)_8_-FITC positive, whereas the percentage of labelled cells raised to more than 90% for the Deca-(Arg)_8_-FITC at the same concentration (Figure 3A). This finding is in accordance with the higher inhibitory effect of Deca-(Arg)_8_ as compared to (Arg)_8_, and strongly support the conclusion that the hydrophobic moiety contributes to a better cell entry of CatLip [36], [39].

### Deca-(Arg)_8_ does not Reduce DHBV Infectivity or PDH Susceptibility to Viral Infection

Because cationic antimicrobial peptides are known to exhibit a potent membrane disruption and antiviral activity, we next asked whether Deca-(Arg)_8_ inhibits DHBV infection either by direct inactivation of DHBV surface or by interaction with either the cellular plasma membrane or the DHBV receptor. Thus, DHBV-positive inoculum was pre-incubated with Deca-(Arg)_8_ prior to PDH infection to analyze the impact on viral infectivity. In a second experiment PDH cells were pre-incubated with Deca-(Arg)_8_ before DHBV infection. However, as illustrated in Figure 3B neither virus pre-treatment with Deca-(Arg)_8_ nor cells pre-incubation with this peptide affected the outcome of DHBV infection.

### Effect of Deca-(Arg)_8_ on DHBV Replication

**Figure 4:**
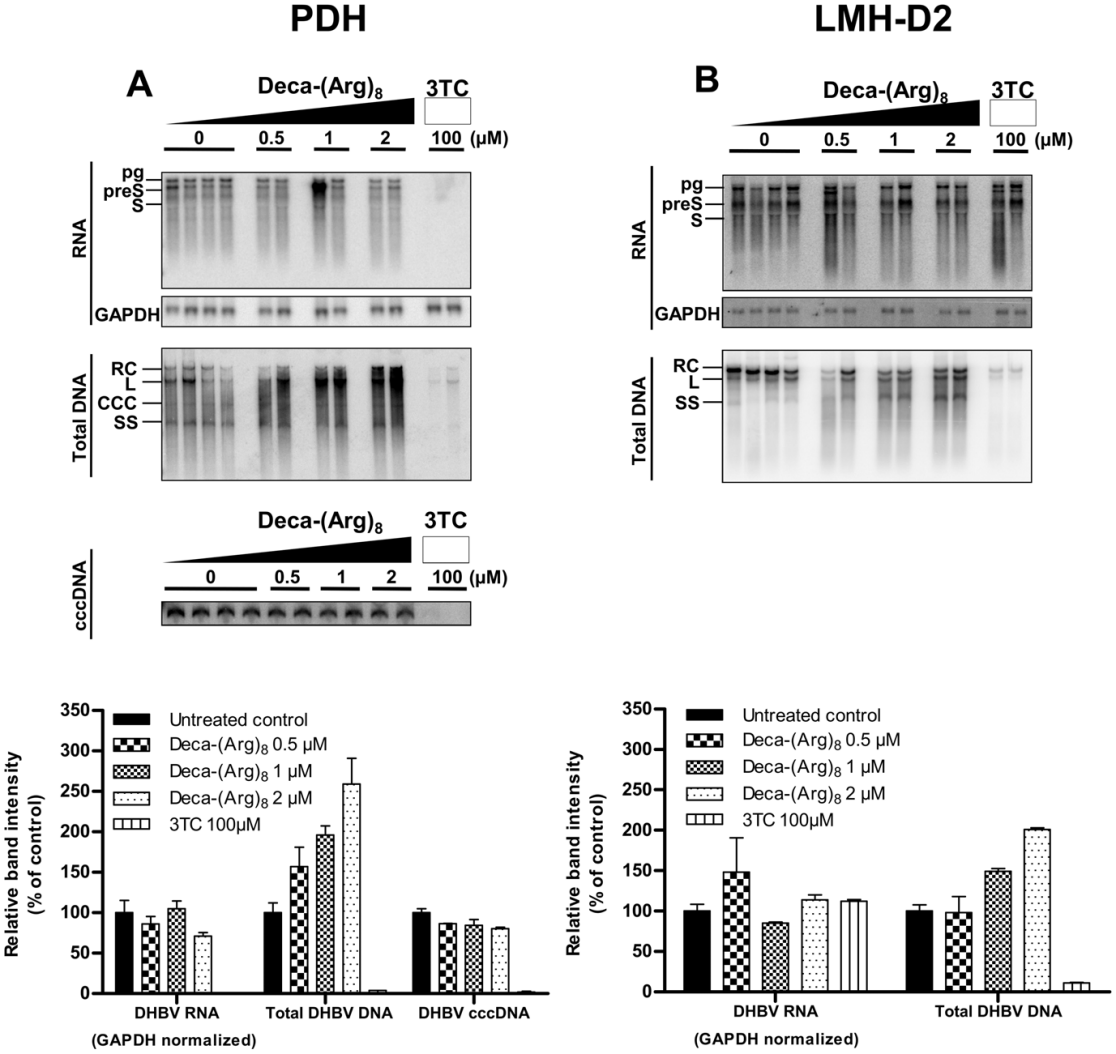
Effect of Deca-(Arg)_8_ on the intracellular hepadnavirus intermediate replicative forms. Infected PDH (**A**), and stably DHBV-transfected LMH-D2 (**B**) cells were treated with different amounts of Deca-(Arg)_8_ ranking from 0.5 µM to 2 µM for 5 days. At the end of treatment, the cells were harvested and lysed for DNA and RNA extraction. Intracellular intermediate replicative forms was subjected to Southern blot and Northern blot analysis with an DHBV probe ^32^P labeled, and was quantified by PhosphorImager scanning using ImageQuant software (Molecular Dynamics). For the Northern blot the level of DHBV RNA was normalized on the GAPDH RNA amount. The top panel shows the autoradiography. The bottom panel shows histograms of autoradiography quantifications. Bands corresponding to the expected size of relaxed circular (RC), linear (L), single-stranded (SS) and covalently closed circular (ccc) DHBV DNA are indicated. Bands corresponding to the expected size of pregenomic (pg), preS (preS), and S (S) DHBV RNA are indicated.

To determine whether the decrease in DHBV secretion is caused by an inhibition of virus replication, LMH-D2 cells and PDHs were treated with different concentration of Deca-(Arg)_8_ followed by quantification of viral RNA and intracellular DNA replication intermediates by Northern and Southern blot, respectively. As a control, cells were treated by 3-TC, which is a specific inhibitor of the DHBV polymerase [32], [33]. As expected 3-TC prevented cccDNA formation in infected PDHs while transfected LMH-D2 cells did not exhibit any detectable cccDNA at all (Figure 4). The latter observation is in accordance with findings of others that transfection of cell lines does not generate significant numbers of cccDNA [40]. As the cccDNA is the template for mRNA synthesis the observation of a 3-TC mediated reduction of viral mRNA synthesis in PDH is also conclusive. An unspecific effect on transcription can be excluded as mRNA synthesis in transfected LMH-D2 cells was not impaired.

Administering Deca-(Arg)_8_ at 2 µM had no significant effect on viral RNA levels in both PDH and LMH-D2 excluding that transcription was affected. However, we observed a dose dependent increase of different viral DNA replicative forms (relaxed circular, linear and single stranded DHBV DNA, Fig. 4A–B), indicating an accumulation of viral replication intermediate-containing capsids. The cccDNA was not affected by Deca-(Arg)_8_ treatment (Fig. 4 A), implying that there is no interference with cccDNA formation by cellular polymerases.

### Effect of Deca-(Arg)_8_ on Viral Structural Proteins Expression and Capsid Formation

**Figure 5:**
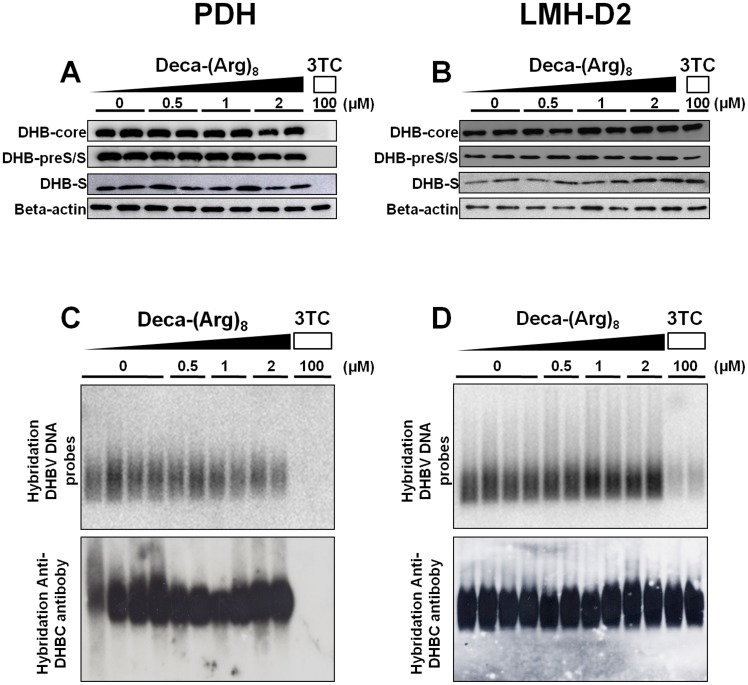
Effect of Deca-(Arg)_8_ peptide treatment on viral structural proteins expression and on intracellular replication-competent core particles formation. Detection of viral structural proteins expression (DHBV-preS/S envelope, DHBV-S envelope and DHBV-core protein) of PDHs (**A**), LMH-D2 cells (**B**) by immunoblotting. After 5 days of treatment by different amount of Deca-(Arg)_8_, cells were harvested and total protein recovered from 12 wells plates was loaded in each lane for SDS-polyacrylamide gel electrophoresis. Following transfer to a PVDF membrane envelope, core protein was detected with specific antibodies. β-actin, detected with mouse anti-human actin, was assayed as a loading control. The next two panels show the results of capsid gel analysis of the native nucleocapsids of PDHs (**C**), LMH-D2 cells (**D**). Core protein was detected by western blotting analysis using a rabbit antiserum reactive to purified DHBV nucleocapsids and the viral DNA was denatured with NaOH and the membrane was probed to detect viral DNA.

We next analyzed the possible impact of Deca-(Arg)_8_ on viral protein expression. Following peptide treatment the expression levels of viral envelope and core protein were not changed neither in PDH nor in LMH-D2 cells (Fig. 5A–B). Core protein assembly also remained unchanged in both cell types as indicated by the migration of capsids in native agarose gels (Fig. 5C–D). Accordingly, DNA intermediates which should be within the capsids were also unaffected by Deca-(Arg)_8_ treatment, in contrast to the control to which 3-TC was added (Fig. 5C–D).

### Deca-(Arg)_8_ Induces Intracellular Envelope Clustering

**Figure 6:**
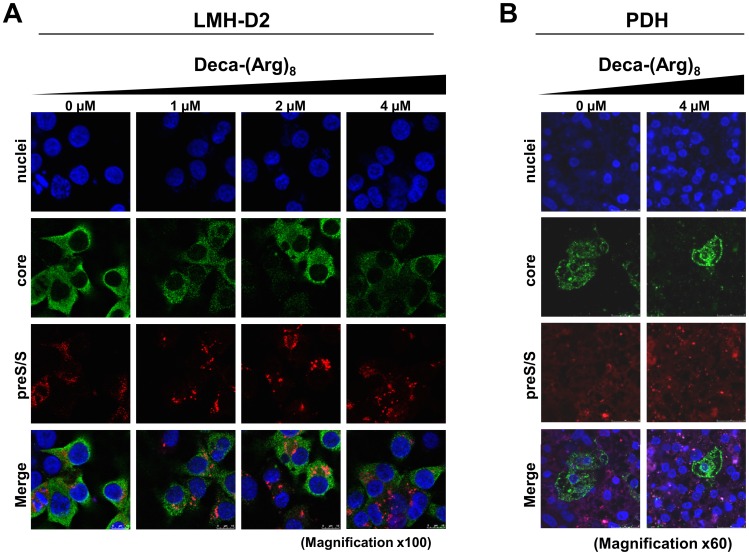
Deca-(Arg)_8_ induces an abnormal distribution and an accumulation of viral envelope proteins in large clusters. LMH-D2 cells (**A**) and DHBV infected PDHs (**B**) were treated with increased concentrations (1–4 µM) of Deca-(Arg)_8_ peptide. Four days after treatment the cells were fixed and immunostained for DHBV-core (rows 2) and DHBV-PreS/S (row 3) using rabbit anti-DHBV-core and mouse anti-DHBV-PreS/S antibodies, respectively. The primary antibodies were followed by staining with AlexaFluor 488-conjugated goat anti-rabbit or AlexaFluor 555-conjugated goat anti-mouse, and the ﬂuorescent signals of DHBV-core (green) and DHBV-PreS/S (red) are shown in corresponding row in absence or presence of Deca-(Arg)_8_ treatment (**A**; columns 1–4) (**B**; columns 1–2). The overlays of the ﬂuorescences are shown in the bottom row (**A–B**). (**A**) Magnificationx100; (**B**) Magnificationx40.

As our data showed that Deca-(Arg)_8_ did not affect DHBV entry, cccDNA formation and protein synthesis we hypothesized that the inhibitory effect was based on virion formation or secretion. First we analyzed the intracellular distribution of viral surface proteins by confocal laser scanning microscopy using indirect immune fluorescence. We investigated the localization of the surface proteins and the core protein/capsids; the latter are indistinguishable by the antibody. Figure 6 illustrates that Deca-(Arg)_8_ treatment led to cluster formation of DHBV preS/S in LMH-D2 and PDH cells (Fig. 6A–B). Image analysis showed that Deca-(Arg)_8_ treatment resulted in a decrease in number of small staining signals while large signals increased (Fig. 6A–B). This effect was more prominent at the higher peptide concentration (4 µM) in both cell types. Following Deca-(Arg)_8_ treatment, 55% of the preS/S staining was localized in structures exhibiting a size larger than 225 µm^2^, whereas in the absence of treatment such large clusters represented only 9% of the total signal (Fig. 6A–B and data not shown).

Core proteins/capsids showed only a partial overlap with the surface proteins. Such partial superposition was expected as secretion and capsid-surface protein interaction depends upon genome maturation inside the capsid [41] and because the majority of the capsids either contained immature DNA or were empty. Core protein/capsid distribution appeared, however, to be unchanged upon Deca-(Arg)_8_ treatment.

### Effect of Deca-(Arg)_8_ on Intracellular Viral Morphogenesis

To examine the effect of Deca-(Arg)_8_ on intracellular viral morphogenesis, we separated viral capsids and surface proteins from cell homogenates on linear iodixanol density-gradients. Analysis was restricted to transfected LMH-D2 cells as PDH cells are not available in sufficient quantities. The quality of the gradient and the presence of intact organelles in the homogenate were controlled in a blot using Rab5B as a marker of early endosomes. As expected the organelle migrated at a low density in lighter fractions of 1.02–1.06 g/mL as previously reported [42], [43].

**Figure 7:**
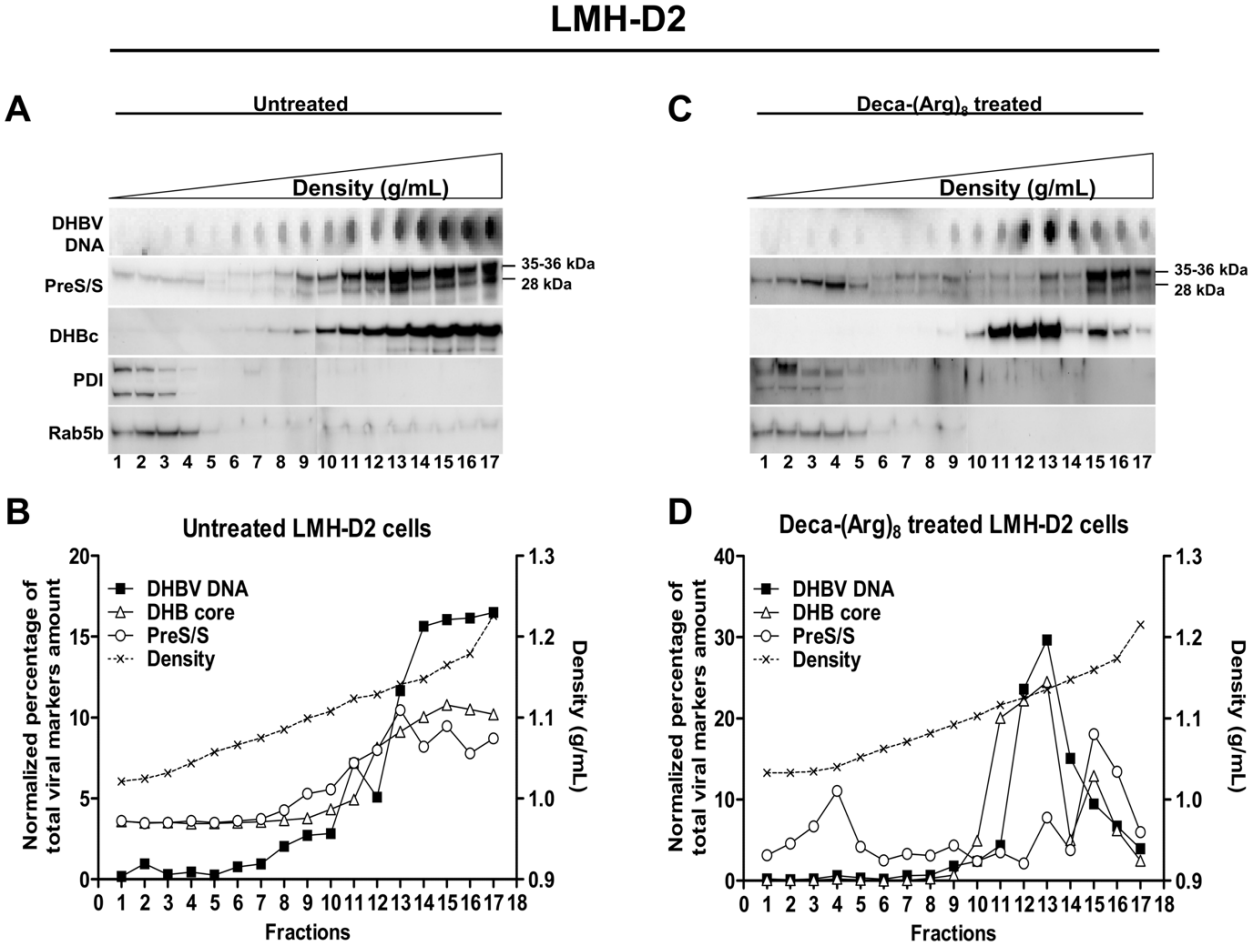
Analysis of subcellular fractions from LMH-D2 for viral and cellular markers. Homogenates of cells were subfractionated using a 0%–30% iodixanol-based linear density gradient and 17 fractions were recovered from top to bottom. Aliquots from untreated cells (**A**) or Deca-(Arg)_8_ treated cells (**C**) of each fraction were separated by 4%–20% gradient SDS-PAGE and analyzed for viral envelope, core protein, and organelle marker proteins PDI (ER), Rab5B (early endosomes). Images of immunoblot were juxtaposed. Viral DNA in the same fractions was analyzed by Dot blot hybridization. In the bottom, the deduced gradient positions and quantification of the viral markers from untreated cells (**B**) or Deca-(Arg)_8_ treated cells (**D**) are represented together with the density profile of the gradient.

In untreated cells (Fig. 7A–B), DHBV DNA and nucleocapsids were concentrated within fractions 13–17 (density 1.14–1.22 g/mL). A number of capsids migrated in slightly lighter factions (fraction 11, density 1.12 g/mL) not superimposing the DHBV DNA or surface protein peaks. This is in agreement with the immune fluorescence data, which implied the presence of a significant number of either immature or empty capsids not attached to the surface proteins. The observed density is in agreement with sedimentation of immature and mature HBV capsids (0.02 g/ml difference) using a similar separation medium [44]. The majority of the preS/S proteins co-migrated with DHBV DNA and capsids but preS/S proteins were also found in lighter factions (fractions 1–5, Fig. 7A–B). These observations showed that the majority of surface proteins were likely either directly attached to capsids or bound to the same cellular compartment. The light fractions migrated similar to PDI. PDI is a marker for the ER [45] and the presence of surface proteins in PDI-containing fractions might indicate the part of preS/S proteins after synthesis which has not already been transported to downstream compartments.

Deca-(Arg)_8_-treatment caused a significant shift of DHBV DNA towards lighter fractions (fractions 11–14; density 1.12–1.14 g/mL) with co-migration of the core protein (fractions 11–13, Fig. 7C–D). PreS/S proteins migrated in the heavier fractions and only a low percentage showed co-migration with viral DNA and capsids. There was, however, another surface protein peak in very light fractions (fractions 1–5) which corresponded to PDI migration. Thus, the large majority of the core proteins and DHBV DNA that sedimented in the fractions 11–13 (with absence of surface proteins) indicates that these fractions were likely naked nucleocapsids.

### Deca-(Arg)_8_ Inhibits the Release of Subviral Particles (SVPs) and Complete Virions

**Figure 8:**
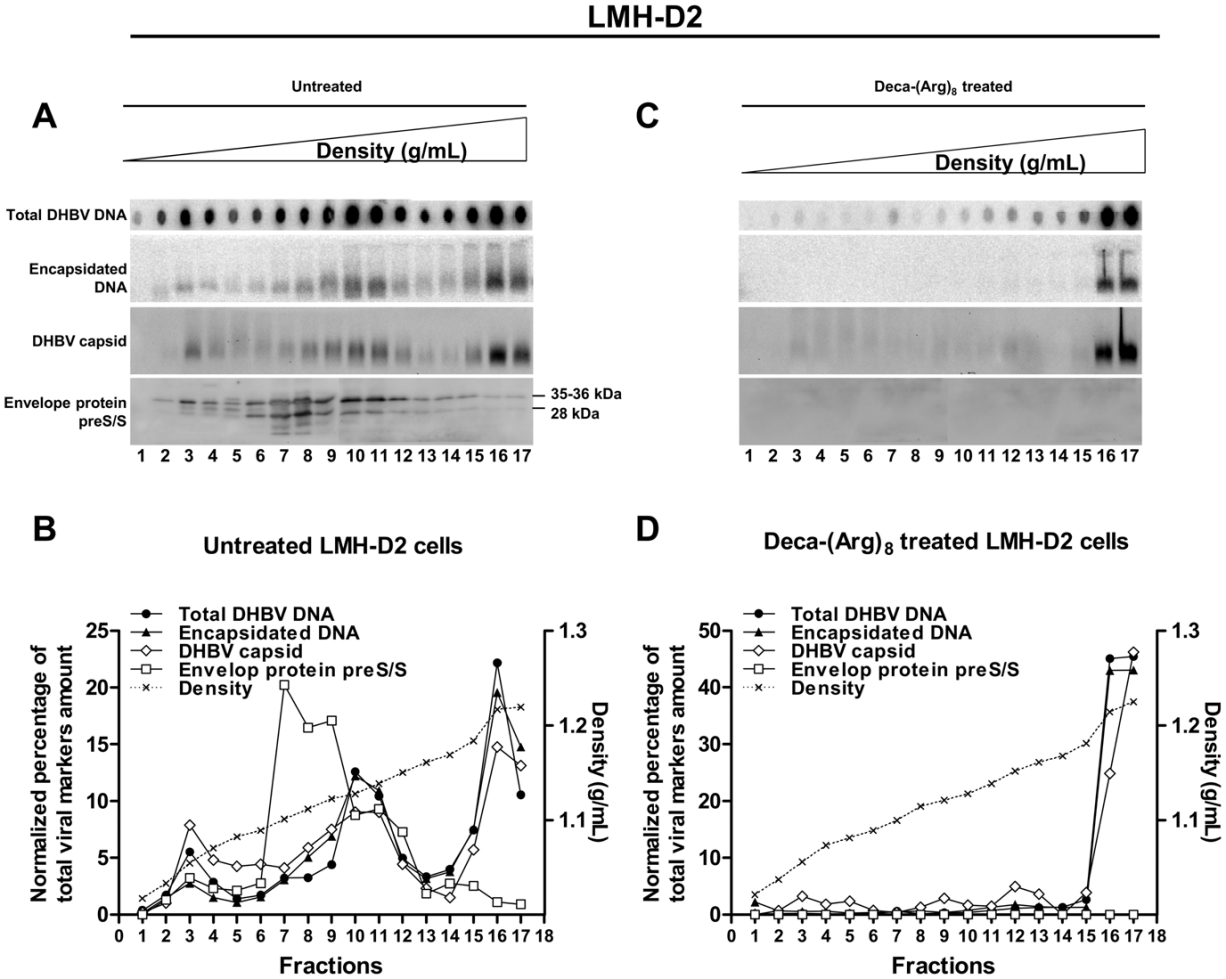
Separation of DHBV DNA-containing particles and SVPs by sucrose-gradient ultracentrifugation. Supernatants of LMH-D2 cells were concentrated and separated using a 0%–60% a sucrose-based linear density gradient and 17 fractions were recovered from top to bottom. Aliquots from untreated cells (**A**) or Deca-(Arg)_8_ treated cells (**C**) of each fraction were separated by 4%–20% gradient SDS-PAGE and analyzed for viral envelope. Images of immunoblot were juxtaposed. DHBV capsids were subjected to electrophoresis through a non-denaturing agarose gel and detected by immunostaining of the viral core protein and viral DNA within nucleocapsids was detected by probing the blots with radiolabeled viral DNA. Total viral DNA in the same fractions was analyzed by Dot blot hybridization. In the bottom, the deduced gradient positions and quantification of the viral markers from untreated cells (**B**) or Deca-(Arg)_8_ treated cells (**D**) are indicated together with the density profile of the gradient.

To investigate if intracellular viral envelope protein clustering by Deca-(Arg)_8_-treatment also has an impact on secretion of the surface proteins in subviral particles and virions, we analyzed LMH-D2 cells supernatants by sucrose gradient centrifugation. In the untreated LMH-D2 cells supernatant DHBV preS/S proteins were detected by Western blot as a major peak within fractions 7 to 12 (1.10 to 1.15 g/mL) (Fig. 8A–B). The fact that the large majority of the preS/S proteins sedimented in fractions 7–9 (with absence of core proteins and DHBV DNA), indicates that these fractions were likely subviral particles. Encapsidated DHBV DNA, indicated by its resistance to nucleases, showed two peaks, one within fractions 10 to 12 (1.13 to 1.15 g/mL) and the second one in fractions 16 to 17 (1.21 to 1.22 g/mL), corresponding to complete virions and most likely to naked nucleocapsids, respectively (Fig. 8A–B). Peaks of DHBV DNA coincided with the migration of DHBV capsids, which were separated on an agarose gel under native conditions (Fig. 8A–B). As illustrated in Figure 8, Deca-(Arg)_8_-treatment completely suppressed the peak of SVPs and virions in fractions 7 to 12 but still showed the peaks of capsids and DNA in the heavy fractions 16 and 17 (1.21 to 1.22 g/mL) corresponding to naked capsids. PreS/S proteins were undetectable throughout the entire gradient implying that Deca-(Arg)_8_-mediated clustering interfered not only with virion but also subviral particle secretion and confirmed that the capsids in fractions 16 and 17 in fact represented naked capsids (Fig. 8C–D). To confirm these findings, LMH cells transfected with a plasmid encoding preS/S protein only, were treated with Deca-(Arg)_8_ at different concentrations. The analysis of the supernatants by immunoblot showed a dose-dependent decrease in preS/S secretion (data not shown).

## Discussion

CPPs and CatLip peptides are interesting ligands for the delivery of (uncharged) biologically active molecules [5], [7], [36]. We unexpectedly found that Deca-(Arg)_8_, belonging to a novel class of drug delivery conjugates designed to improve the bioavailability of their PNA cargo [36], was able to drastically inhibit DHBV and HBV release in a dose-dependent manner. Inhibition depended upon the length of the lipid moiety and the number of positively charged arginine residues. Strongest inhibition was observed for Deca-(Arg)_8_. (Arg)_8_ also exhibited an inhibitory effect, but this was less pronounced supporting the conclusion that the hydrophobic moiety contributes to a better cell entry of CatLip [39]. The arginine composition of our active compounds are in accordance with earlier substitution assays, showing that arginine give stronger HSV antiviral properties than lysine [46], [47]. The lower inhibitory effect of (Arg)_8_ compared to Deca-(Arg)_8_ was consistent with a lower uptake using PDHs. These cells were chosen, as they are the natural targets for DHBV infection.

However, even at the highest concentration tested, only 90% of the PDHs became positive for Deca-(Arg)_8_-FITC. This observation is in agreement with the 90% inhibition of DHBV DNA secretion. As we showed that Deca-(Arg)_8_ acts on viral morphogenesis, these data also support that Deca-(Arg)_8_-FITC was internalized into the cells and not only attached to cell surface. The observation that not all cells become FITC-positive argues against passive diffusion but signifies active uptake as it has been reported [3], [4], [7]. The finding further indicates that there are inter individual differences even in cell culture, which is in agreement with observation of others analyzing uptake of viruses in cultured cells [48], [49].

In total three different pathways of CPP action are suggested in the literature [10]: by activation of innate cellular response (HIV, HSV), direct interaction with the virus (VSV, HSV and HIV) or interference with viral uptake via interaction with cellular receptors (HSV, HIV, and HCMV). Our findings that Deca-(Arg)_8_ pretreatment of virus had no inhibitory effect implies that Deca-(Arg)_8_ does not change the DHBV surface proteins in a way that interaction with the receptor or internalization becomes inhibited. As hepadnaviruses are sensitive to structural changes of the surface as for instance shown by disulfide bridge disruption or lipid removal [16], it is likely that Deca-(Arg)_8_ does neither interact with the viral surface proteins nor with the integrated lipids. Similarly pretreatment of PDHs with Deca-(Arg)_8_ did not interfere with DHBV infection supporting that the unknown DHBV receptor was not the target of the inhibitor in contrast to for example the interaction of glycoaminoglycans with HSV [50], [51]. It further suggests that Deca-(Arg)_8_ did not act via stimulation of innate immunity.

Analyzing the impact of Deca-(Arg)_8_ on viral mRNA synthesis and intracellular genome maturation we used 3-TC as control. 3-TC is a nucleoside analogue inhibiting exclusively the viral polymerase at the used concentration [32], [33]. Our observations indicate that Deca-(Arg)_8_ does not block reverse transcription in contrast to 3-TC. Moreover, Deca-(Arg)_8_ had no impact on all steps required for progeny capsid formation including mRNA synthesis and on retrograde transport, which occurs via microtubules [17], which is also in agreement with the lacking effect of Deca-(Arg)_8_ on mRNA synthesis in LMH-D2 cells. The observed accumulation of replication intermediates in Deca-(Arg)_8_-treated cells thus implies that the viral capsids comprising the replication intermediates were not able to leave the cells.

Native agarose gel electrophoresis demonstrated that the capsids from Deca-(Arg)_8_-treated cells migrated identical to those of the untreated control. This finding demonstrates that there was no change of the capsids surface charge due to Deca-(Arg)_8_ attachment as the surface charge is the main physical property determining protein migration in native gels. The observation thus means that a restriction of capsid escape from the cells was likely neither due to a changed structure of the capsids nor to a loading of capsid surface with Deca-(Arg)_8_.

The physiological capsid exit of the cells requires virion formation driven by an interaction between the mature capsid and the preS domain of the surface proteins [22], [52]. PreS has thus to be partially exposed to the cytoplasmic side. In fact preS shows a dual topology with some domains exposed to the exterior of the virus (luminal side of the ER) and to the inner side of the virus (corresponding to the cytosolic exposure) [22], [53]. It was suggested that the preS domain can switch its orientation by a mechanism, which is not known [53]. Our observation that Deca-(Arg)_8_ treatment causes cluster formation may suggest severe structural changes of preS/S, which may prevent interaction with the capsid. In fact, gradient centrifugation of cell culture supernatants and cell lysates from LMH-D2 cells confirmed that Deca-(Arg)_8_ treatment inhibited virion formation and secretion, although intracellular preS/S synthesis was not impaired. In accordance with these findings we observed that in Deca-(Arg)_8_-treated cells, subviral particles were no more secreted although they use a different secretion pathway compared to complete virions [23], [52], [54]. However, the observation that naked capsids were still released corroborated the conclusion that the primary target of Deca-(Arg)_8_ is the proper formation of preS/S multimers prior to virion formation. The inhibition of subviral particle and virion release could be explained by the abnormal distribution and the accumulation of preS/S envelope proteins in large clusters observed under Deca-(Arg)_8_ treatment. Indeed, like other typical membrane proteins, the DHBV envelope proteins are synthesized within the endoplasmic reticulum and gain into a relatively complex topology, with four transmembrane domains required for proper folding, being essential for recruitment of a mature viral nucleocapsids, nucleocapsid envelopment, SVPs formation and virion/SVP budding [22]. As CPPs interact directly with cellular membranes and destabilize the lipid bilayer [3], it can be suggested that Deca-(Arg)_8_ by interacting with different intracellular membranes interferes with the formation of virions and SVPs and by the fact their release. Although we do not know if Deca-(Arg)_8_, exhibits a specificity for one specific intracellular membrane it appears likely that such interference may change surface protein conformation and/or availability (*e.g.* sequestration in a compartment) leading to inhibition of SVP and virion formation and release. In conclusion our data are consistent and provide new highlights to the recent observations [23], [26] demonstrating that the egress of naked hepadnaviral capsid implies a non-vesicular exocytosis process, unlike complete virions and subviral particle release pathways.

Taken together, our data provide the first evidence that a cationic lipopeptide, Deca-(Arg)_8_, is able to dramatically decrease the release of DHBV virions and subviral particles from infected cells by alteration of late stages of DHBV morphogenesis, without affecting naked viral capsid release. Since Deca-(Arg)_8_ does not target the viral polymerase, responsible for the emergence of resistant mutants, it may represent a potentially interesting compound for the development of new inhibitors against chronic hepatitis B, although the inhibitory activity of such CatLip peptides against human HBV needs to be further investigated. In this regard, our preliminary data indicate already the dose-dependent inhibition of HBV release in Deca-(Arg)_8_-treated HepG2.2.15 cells. Moreover, because the core antigen is extremely immunogenic, the secretion of large amounts of naked nucleocapsids following Deca-(Arg)_8_ treatment may be of particular value for immune response stimulation in chronic HBV carriers. Finally, since Deca-(Arg)_8_ inhibits the secretion of both subviral particles and virions, but does not interfere with naked nucleocapsid egress, it could represent a valuable tool for better understanding of hepadnaviral morphogenesis mechanisms and in particular their export pathway, which is far from being fully elucidated.

## Methods

### Synthesis of Cell-penetrating Peptides

The sequences of the CPPs are listed in Table 1. CPP synthesis was carried out using standard Fmoc-solid phase chemistry, and the peptides were purified by RP-HPLC and characterized by MALDI-TOF mass spectrometry [6], [39].

### Virus

A pool of viremic sera from ducklings infected with the cloned and sequenced DHBV was used as an inoculums [55]. This inoculum was quantified into virus genome virus equivalents (vge) by quantitative dot blot hybridization as previously described [28].

### Cell Culture

Primary duck hepatocytes (PDH) were isolated from Pekin duck embryos as previously described [56]. DHBV infection was performed at the same time as plating by overnight incubation (16 h) of the hepatocytes with a virus-positive duck serum (30 virus genome equivalents (vge) per cell). The previously described avian hepatoma DHBV transfected LMH-D2 cell line [37], [38] was grown as previously described [57]. The stably HBV-transfected cell line HepG2.2.15 was grown as previously described [58]. Different amounts of CatLip were added daily to the culture medium from day 1 post-seeding and cells were lyzed at the end of treatment for analysis of intracellular viral DNA, RNA, and proteins. The growth medium was harvested and changed daily. The cellular toxicity was analyzed by daily examination of cells with a light microscope and by a cell toxicity test based on the (3-(4,5-dimethylthiazol-2-yl)-2,5 diphenyl-tetrazolium-bromide (MTT) assay.

### Analysis of DHBV DNA

Cells supernatants were harvested daily, spotted onto positively charged nylon membrane and extracellular DHBV DNA was detected in supernatant of cells by dot blot hybridization assay using a full-length ^32^P-labelled DHBV probe as described previously [28]. For DHBV intermediate replicative forms analysis, DNA was extracted from cells and 10 µg of the total cellular DNA were subjected to electrophoresis on 1% agarose gel, followed by Southern blot analysis and hybridization with ^32^P-labeled genomic DHBV probe as previously described [28]. Viral DNA was quantified by PhosphorImager scanning using ImageQuant software (Molecular Dynamics).

### Antibodies and Plasmids

DHBV-preS/S and DHBV-core proteins were detected using previously described rabbit polyclonal antibodies [59], [60]. DHBV-S protein was detected with a mouse monoclonal antibody [61]. β-actin, Rab5b and protein disulfide isomerase (PDI) was detected with primary polyclonal antibodies (Santa Cruz Biotechnology). The construction of plasmids pCI-preS/S was performed as described previously [27], [62].

### Analysis of Intracellular Replication-competent Core Particles

Intracellular replication-competent core particles were purified following the protocol described by Ren and Nassal [63]. To release intracellular cores, cells were resuspended in lysis buffer (50 mM Tris-HCl [pH 8.0], 10 mM EDTA, 1% NP-40) and incubated at 37°C for 15 min. Nuclei and cellular debris were removed by centrifugation. Mg^2+^acetate (final concentration, 10 mM) and DNase I (final concentration, 500 µg/ml) were added to the supernatants and the mixture was incubated at 37°C for 45 min to digest nonencapsidated DNA. For native agarose gel electrophoresis, 10% of the lysate was loaded on a 1% agarose gel. The gel was blotted on a positively charged nylon membrane or a hydrophobic polyvinylidene difluoride (PVDF) membrane (Amersham Hybond™-N+ and Hybond™-P) by capillary transfer in TNE buffer (10 mM Tris-HCl [pH 7.5], 150 mM NaCl, 1 mM EDTA). The nylon membrane was soaked in 0.5 M NaOH–1.5 M NaCl and neutralized in 0.2 M Tris-HCl; (pH 7.5)–1.5 M NaCl (5 min each) and was fixed by heating at 80°c for 1 h. Viral specific nucleic acids were detected with a ^32^P labeled probe of full-length linear viral genome. Core protein was detected on the PVDF membrane using the rabbit polyclonal anti-DHBc antiserum and a peroxidase-conjugated secondary antibody with a chemiluminescent substrate (ECL-Plus, Amersham).

### SDS-PAGE and Immunoblotting

Cells were lysed in a TNE buffer supplemented with 0.5% of Triton X100. Cellular debris were removed by centrifugation (10,000 g-2 min). An aliquot of each sample (10 µg) was denaturated with 1/5th volume of 5×Laemmli buffer and boiled for 5 min. The samples were separated by in a 12% acrylamide SDS-PAGE and subsequently proteins were transferred onto PVDF membranes. Following blocking with 10% dried milk diluted in Phosphate-buffered saline 1X (PBS), the membrane was incubated for 2 h at room temperature (RT) or overnight at 4°C with anti-DHBV proteins antibodies. After 3 washings with PBST (PBS, tween 0.1%), viral proteins were detected using a peroxidase-conjugated secondary antibody (at a dilution of 1∶5,000) with a chemiluminescent substrate (ECL-Plus; Amersham). The S and pre-S antigens were revealed in the supernatants of transfected LMH, LMH-D2 or PDH cultures by the same method as described above. The immunoblot signals were quantified using a chemiluminescence imager (BioRad).

### Subcellular Fractionation and Iodixanol Gradient Ultracentrifugation

LMH-D2 cells were plated into 150 mm dishes at a density of 1.10^7^ cells per dish and were treated daily with 2 and 4 µM of Deca-(Arg)_8_ respectively. At the end of treatment plates were washed twice with homogenization buffer (HB) (0.25 M sucrose, 1 mM EDTA, 60 mM HEPES pH 7.4, and protease inhibitor cocktail; Roche) and harvested by gentle scraping into 2.5 ml of homogenization buffer. Subcellular fractionation was performed following iodixanol gradient ultracentrifugation as described by Mhamdi *et al.* [43]. The suspensions were centrifuged for 4 minutes at 100 g and 4°C, and then the pellets were washed twice with HB. Afterwards, the pellets were resuspended in 0.5 mL HB and were homogenized it in a glass homogenizer for 20 strokes. The homogenates were centrifuged for 10 minutes at 2,500 g and 4°C and the postnuclear supernatants (PNS) were transferred into a new tube. The pellet was resuspended in 500 µL HB, recentrifuged and the obtained PNS were pooled with the first PNS. For a 0%–30% linear iodixanol gradient, we initially diluted a stock solution of 60% (wt/vol) Iodixanol (optiprep, Sigma-Aldrich) to 30% by adding 1 volume of Optiprep to 1 volume of the HB solution. With this working solution, 12 mL of discontinuous density step gradients (5, 10, 15, 20, 25 and 30% iodixanol in HB) were prepared. The PNS were loaded on top of the gradient and centrifuged for 2 hours at 288,000 g and 4°C in a Beckman SW Ti 41 rotor, followed by 17 fractions collection.

### Indirect Immune Fluorescence Detection of Viral Antigens by Confocal Laser Scanning Microscopy

LMH-D2 and PDH cells were plated into glass chambers and were treated with different amount of Deca-(Arg)_8_ peptide daily during 4 days. Cell preparation and immune staining for DHBV-core and DHBV-PreS/S was performed as previously described [58]. The cells on the cover slips were analyzed by confocal laser scan microscopy using a Leica SP5 LSM and a 63×lens. Images were taken at a pinhole of 1 and the filter settings for the corresponding fluorophore. The recorded images were analyzed by ImageJ software and statistical analysis was performed using Excel. Figures of the images were arranged using Adobe Photoshop.

### Extracellular DHBV Particles Purification and Sucrose Gradient Ultracentrifugation

LMH-D2 cells were plated into 150 mm dishes at a density of 1.10^7^ cells per dishes and were treated daily with 2 µM of Deca-(Arg)_8_ for 4 days as described above. Supernatants were centrifuged at 8,000 *g* for 15 minutes at 4°C, filtered through 0.45-µm membranes and ultracentrifuged at 25,000 g for 4 hours at 4°C. Pellets were then resuspended in 1 mL of TNE, layered at the top of a 10% to 60% sucrose linear gradient, and submitted to isopycnic ultracentrifugation for 16 hours at 45,000 g at 4°C. 17 fractions (700 µL each) were then collected from the top of the gradient. Fractions were analyzed by immunoblot for viral proteins and Dot blot hybridization assay for DHBV DNA.

## Supporting Information

Figure S1 **Dose dependent inhibition of hepadnaviral release by Deca-(Arg)_8_ in HBV-transfected HepG2.2.15.** Stably HBV-transfected HepG2.2.15 cells were treated with different amounts of Deca-(Arg)_8_ ranking from 0.5 µM to 4 µM in duplicates for 5 days. Cell culture supernatants were collected daily during treatment. The kinetics of viral release in cell culture supernatants, monitored by dot-blot hybridization and quantified by PhosphorImager scanning using ImageQuant software (Molecular Dynamics), is represented on the upper panel. Relative areas under curves determined by the means of duplicates, compared to untreated cells, are represented in the lower panel. The curves are representative of at least two independent experiments.
(TIF)

Figure S2 **Effect on hepadnaviral release of (Arg)_8_ and Decanoic acid treatment in different cell culture systems.** DHBV infected PDH (**A**); stably DHBV-transfected LMH-D2 (**B**) were treated with different amounts of (Arg)_8_ and decanoic acid ranking from 0. 5 µM to 10 µM in duplicates for 6 and 4 days respectively. Cell culture supernatants were collected daily during treatment. Viral release in cell culture supernatants was monitored by dot-blot hybridization and was quantified by PhosphorImager scanning using ImageQuant software (Molecular Dynamics). Relative areas under curves determined by the means of duplicate, compared to untreated cells, are represented.
(TIF)
